# Validation of semi‐automated anatomically labeled SEEG contacts in a brain atlas for mapping connectivity in focal epilepsy

**DOI:** 10.1002/epi4.12499

**Published:** 2021-05-15

**Authors:** Kenneth N. Taylor, Anand A. Joshi, Tugba Hirfanoglu, Olesya Grinenko, Ping Liu, Xiaofeng Wang, Jorge A. Gonzalez‐Martinez, Richard M. Leahy, John C. Mosher, Dileep R. Nair

**Affiliations:** ^1^ Epilepsy Center Neurological Institute Cleveland Clinic Cleveland OH USA; ^2^ Department of Electrical Engineering University of Southern California Los Angeles CA USA; ^3^ Department of Pediatric Neurology Gazi University School of Medicine Ankara Turkey; ^4^ Hauenstein Neuroscience Center Mercy Health Grand Rapids MI USA; ^5^ Department of Neurological Surgery and Epilepsy Center University of Pittsburgh Medical Center Pittsburgh PA USA; ^6^ Department of Neurology McGovern Medical School University of Texas Health Science Center at Houston Houston TX USA

**Keywords:** anatomical labeling, epilepsy, SEEG

## Abstract

**Objective:**

Stereotactic electroencephalography (SEEG) has been widely used to explore the epileptic network and localize the epileptic zone in patients with medically intractable epilepsy. Accurate anatomical labeling of SEEG electrode contacts is critically important for correctly interpreting epileptic activity. We present a method for automatically assigning anatomical labels to SEEG electrode contacts using a 3D‐segmented cortex and coregistered postoperative CT images.

**Method:**

Stereotactic electroencephalography electrode contacts were spatially localized relative to the brain volume using a standard clinical procedure. Each contact was then assigned an anatomical label by clinical epilepsy fellows. Separately, each contact was automatically labeled by coregistering the subject's MRI to the USCBrain atlas using the BrainSuite software and assigning labels from the atlas based on contact locations. The results of both labeling methods were then compared, and a subsequent vetting of the anatomical labels was performed by expert review.

**Results:**

Anatomical labeling agreement between the two methods for over 17 000 SEEG contacts was 82%. This agreement was consistent in patients with and without previous surgery (*P* = .852). Expert review of contacts in disagreement between the two methods resulted in agreement with the atlas based over manual labels in 48% of cases, agreement with manual over atlas‐based labels in 36% of cases, and disagreement with both methods in 16% of cases. Labels deemed incorrect by the expert review were then categorized as either in a region directly adjacent to the correct label or as a gross error, revealing a lower likelihood of gross error from the automated method.

**Significance:**

The method for semi‐automated atlas‐based anatomical labeling we describe here demonstrates potential to assist clinical workflow by reducing both analysis time and the likelihood of gross anatomical error. Additionally, it provides a convenient means of intersubject analysis by standardizing the anatomical labels applied to SEEG contact locations across subjects.

**Key Points**

- The ability to precisely determine the anatomical location of SEEG electrode contacts is critical for both medical diagnosis and research
- Automated labeling has applications in clinical workflow optimization and training

## 1 INTRODUCTION

The SEEG methodology, originally introduced by Talairach and Bancaud in the 1960s, involves implanting depth electrodes under stereotaxic guidance to allow epileptologists to record intracranial EEG (iEEG) activity from various cortical and subcortical structures.^1^, ^2^ This methodology provides the ability to record both interictal and ictal data from within cortical regions of the epileptic network in a given patient and is often used to explore complex cases of focal epilepsy such as those patients who have a nonlesional MRI or in whom noninvasive data are poorly concordant in localizing the epileptogenic zone.^3^ The principle of SEEG involves analysis of iEEG data, both during interictal and ictal periods, in relation to the anatomical and clinical features. This type of analysis performed during SEEG is termed the anatomical‐electroclinical correlation.^4^ Interpretation of the iEEG data from SEEG requires a clear understanding of the three‐dimensional anatomy covered by depth electrodes. These electrodes, each consisting of up to 16 individual contacts, can be placed in a variety of different trajectories which further complicates the anatomical correlation. Traditionally, implantation orthogonal to the sagittal plane is preferred by most epileptologists as a way to reduce the complexity of anatomical coverage. However, there are instances when an oblique trajectory is used in order to maximize coverage of deep structures such as the insula or the posterior portion of the gyrus rectus. It is not uncommon for a single depth electrode to cover several anatomic structures. For example, an electrode placed orthogonally into the lateral temporal lobe could pass through three distinct anatomic structures: superior temporal gyrus, Heschl's gyrus, and posterior long gyrus of the insula. The subsequent analysis of an ictal discharge arising from one of these regions with varying propagation patterns can make the iEEG difficult to interpret whether an accurate anatomical localization of each contact on the depth electrodes is not well understood.^3^ Further complication of the anatomical analysis arises due to interictal and ictal propagation which can spread via white matter association fibers.^5^ As a result, the localization of epileptic activity may not be restricted to strictly adjacent structures. Careful analysis of propagated activity also requires a clear understanding of the underlying anatomy covered by the intracranial electrode.

Multiple factors play a role in the successful analysis of intracranial data as it relates to the accurate anatomic localization of implanted SEEG electrodes, particularly the three‐dimensional anatomy. Normal anatomy can be distorted by pathology or surgery. For example, a case of periventricular nodular heterotopia and epilepsy studied by SEEG showed that the epileptogenic zone involved the periventricular nodule and adjacent white matter and not the overlying cortex.^6^ A careful review of this complex anatomo‐electro‐clinical correlation leads to a successful surgical ablation of the epileptogenic zone that has rendered the patient seizure free now for three years. A clear understanding of the anatomical location of the SEEG electrodes allowed for clear localization of the complex anatomo‐electroclinical correlation in this case.

Another reason to understand anatomical localization of electrodes is that epileptiform activity can propagate within several milliseconds to remote cortical regions from its onset site.^5^ It has been widely accepted that focal epilepsy manifests as a network disorder.^7^, ^8^, ^9^ Mapping the effective connectivity of cortical sites involved in the ictal and interictal epileptiform activity recorded during SEEG is important in making sense of the data.^10^ Propagated activity through large‐scale brain networks can complicate SEEG interpretation. Interareal connectivity can be investigated using a technique called cortico‐cortical evoked potentials (CCEPs).^11^, ^12^ Studies of connectivity measures across large datasets also require consistent labeling of contact locations. The ability to accurately group large sets of patients based on similar anatomo‐clinical electrical correlations has a variety of applications. Computing propagation metrics between electrode pairs using the CCEP paradigm yields directional signal networks which can be used to investigate their relation to the location of the epileptogenic zone.^13^ For example, connectivity analysis of a large CCEP dataset covering the insula reveals varying connectivity patterns from each insular gyrus to various large‐scale cortical regions.^14^ A standardized method of anatomical labeling which is consistent in its approach is desirable for such data analysis.

Methods have been developed to accomplish automated anatomical labeling, particularly for subdural grids. Qin et al^15^ studied localization for both SEEG and electrocorticography (ECoG) contacts on pre‐operative T1 MRI and postoperative CT. Validation of the SEEG electrode localization was performed based on analysis of intraoperative photographic images. As noted by the authors, this approach cannot fully validate all contact locations as only superficial positions can be photographed. Granados et al^16^ reported a method of automatically segmenting SEEG contacts relative to the anatomy while accounting for electrode bending. The authors found strong agreement when identifying the anatomical region of the brain at the tip of the electrode between their algorithm and manual assignment by two neurosurgeons. Another study comparing several methods of localizing subdural electrodes to overlying gyri found that a recursive grid partitioning method was most accurate.^17^ Important clinical and research‐related requirements for precise and rapid labeling methodology for anatomical localization of SEEG depth electrodes are also described in Refs 15, 18.

In this paper, we present a semi‐automated methodology for labeling SEEG electrodes according to the underlying subject anatomy. We compare the labeling produced by the semi‐automated method to a large set of labels manually assigned to SEEG contacts by trained clinical epilepsy fellows. We describe a methodology which provides a solution to the problem of standardizing anatomical labeling for large datasets. The process requires a pre‐operative MRI and postoperative CT and allows the user not only to obtain anatomical labels for each individual SEEG electrode contact, but also to map the contacts to a common atlas space for group analysis. The method is validated via comparison of the labels against those manually assigned during our standard clinical workflow. Cases of disagreement of labels for the two methods were reviewed by two experts as we report below.

## 2 MATERIALS AND METHODS

### 2.1 Subjects

Data were analyzed from patients who underwent SEEG during surgical evaluation for medically intractable focal epilepsy. Subjects were implanted with SEEG depth electrodes as a part of routine clinical assessment of suitability for surgical intervention. We examined 109 consecutive subjects in our cortico‐cortical evoked potentials (CCEPs)/SEEG database from 2016 to 2017. Six of the 109 patients were excluded from the analysis due to incompatibility with the proposed method (4 coregistrations done with only FLAIR MRI sequence, 1 with 7T MRI, and 1 with incomplete data).

### 2.2 Clinical evaluation

The need for SEEG evaluation was determined during patient management conference. One of the main indications for SEEG is to explore a surgical hypothesis when the noninvasive datasets are not sufficiently concordant to localize the epileptogenic zone.^4^ Anatomo‐electro‐clinical hypotheses were formulated individually for each patient during a multidisciplinary patient management conference based on available noninvasive data: clinical history, video EEG, MRI, PET, ictal SPECT, and MEG. SEEG implantation was performed using multilead depth electrodes (AdTech, Racine, Wisconsin; Integra, Plainsboro, New Jersey; or PMT, Chanhassen, Minnesota). The SEEG procedure is performed under general anesthesia using a stereotactic frame. Depth electrodes were placed using trajectories that were either orthogonal or oblique to the sagittal plane, based on the pre‐implantation planning.^19^

The positions of depth electrodes were checked by the digital fusion of a postimplantation thin‐sliced CT 3D image with a pre‐operative T1‐weighted volumetric MRI. Images were aligned using the CURRY 7 software (Compumedics NeuroScan, Hamburg, Germany). Clinical fellows manually labeled the anatomical locations of each individual electrode, in a process which typically takes 3‐4 hours. These epilepsy fellows were in their 7th year of postgraduate medical training, and their anatomical labelings were supervised by staff neurophysiologists involved in the interpretation of the SEEG clinical results. In this study, we examined the resulting set of all clinical labels for a large cohort of patients, adjusting the labels for consistency in terminology, spelling, or abbreviations used by the fellows. Further, we then excluded any label which was not present in at least three subjects, which would suggest an atypical anatomic terminology. The result was a “harmonized” set of 167 “clinical labels” that had been assigned to contacts in a least three patients in a cohort of 103 patients.

### 2.3 Semi‐automated SEEG contact labeling

Each patient's MRI was processed using BrainSuite^20^ to coregister the brain volume to the USCBrain atlas.^21^ BrainSuite uses a cortically constrained and nonrigid volume registration method^22^ to align a subjects’ MRI to that of the atlas. The region boundaries and labels from the atlas are then propagated back to the subject to label individual anatomical structures. For visualization purposes, so that the labeled brain volume and SEEG electrodes can be viewed in a common space, the subjects’ MRIs and labeled brain volumes were then imported into Brainstorm.^23^

The USCBrain atlas was constructed using both anatomical and functional information to guide the parcellation of the cerebral cortex.^24^ First, a single‐subject, high‐resolution T1‐weighted image was extracted and anatomically labeled manually on coronal single‐slice images guided by sulcal and gyral landmarks to generate the BCI‐DNI atlas, containing 66 cortical (gyral) regions and 29 noncortical regions. The gyral regions were then further subparcellated using resting fMRI data from multiple (n = 40) subjects from the Human Connectome Project (HCP) database. All subjects’ fMRI data were coregistered to the common atlas space, and then, each gyrus was either left as is or subdivided into as many as four subdivisions based on differences in resting‐state functional connectivity patterns across the gyrus. The number of subdivisions depended on the number of distinct connectivity patterns that could be found consistently across the 40 subjects within that gyrus.^25^ The resulting USCBrain atlas has a total of 130 cortical regions and 29 noncortical regions. The atlas also includes an optional subdivision of each gyrus that delineates the sulcal valleys from gyral crowns for each of the 26 primary on each cortical hemisphere.^24^

As discussed above, during the clinical evaluation Curry 7 was used to register the CT image to the MRI and identify the contact locations. To translate these 3D locations into the Brainstorm coordinate system, we first extracted from Curry the array of electrodes and the corresponding scalp tessellation generated by Curry, both registered in the Curry coordinate system. In Brainstorm, the exact same patient MRI was imported, and Brainstorm generated the same scalp tessellation, albeit in Brainstorm coordinates. We then automatically aligned the Curry scalp tessellation with the Brainstorm scalp tessellation, thereby bringing the two different coordinate systems into agreement and aligning the corresponding 3D contact locations within the Brainstorm MRI. An obvious alternative approach would have been to do the CT registration and contact identification completely within BrainSuite and Brainstorm, without relying on Curry. A possible confound of this approach would have been differences in the software registration methods between the manual and semi‐automated labeling methods that we are comparing. By using the above approach, we ensure that atlas‐based labeling used the same CT registration from Curry that the fellows had used in their clinical labeling. In this way, we guaranteed that the electrode contacts are in identical locations with respect to subject MRIs for both methods.

With the electrodes coregistered to the MRI‐based anatomy in the Brainstorm coordinate system, anatomical labels from the USCBrain atlas were then assigned to each contact. Each contact was initially localized to a single point in 3D space, when in reality they were approximately 2 mm in length and 0.8 mm in diameter. To account for this physical size and to better determine whether a contact lay on an anatomical boundary with respect to the atlas, we constructed a 2‐mm‐radius sphere around each contact represented by a set of 32 points on the surface of the sphere (utilizing Brainstorm's built‐in SEEG visualization tools). The SEEG contacts were assigned labels according to the regions in the USCBrain atlas in which the center of the contact lay. Labels determined using this process are referred to below as “USCBrain labels.” We also describe below how the spherical representation of each contact was used in comparing atlas‐based and manual clinical labeling.

**FIGURE 1:**
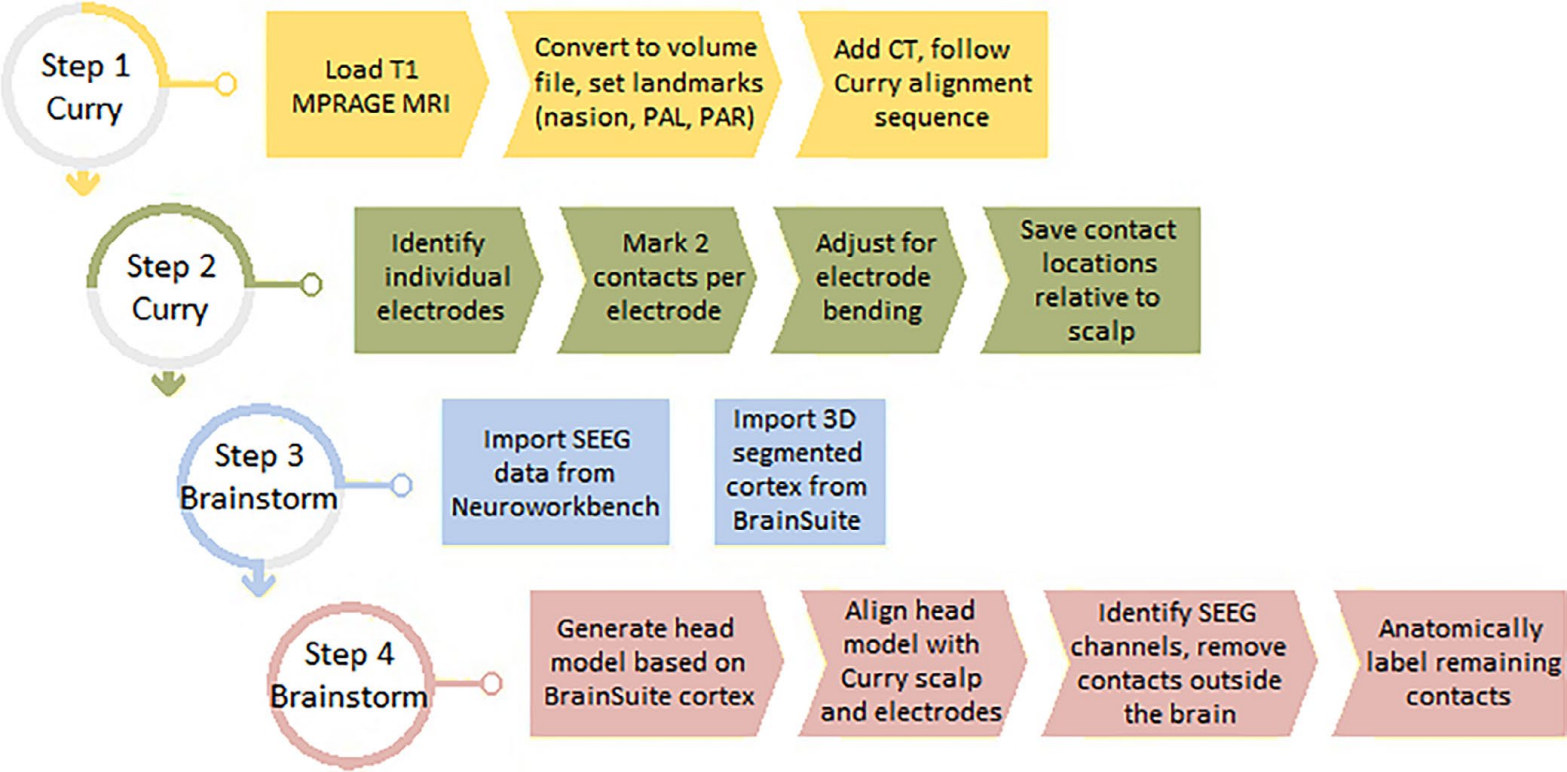
Diagram of all steps in the anatomic labeling process. The MRI and CT are aligned within Curry. Individual electrodes are then identified, and 2 contacts on each are marked. With a known number of contacts and spacing given by the model, this tracks the trajectory of each electrode. Manual refinement of each electrode then corrects for bending during insertion. The resulting coordinates can then be combined with the BrainSuite‐segmented cortex and SEEG data into an automated Brainstorm process which anatomically labels the SEEG data contact by contact

**FIGURE 2:**
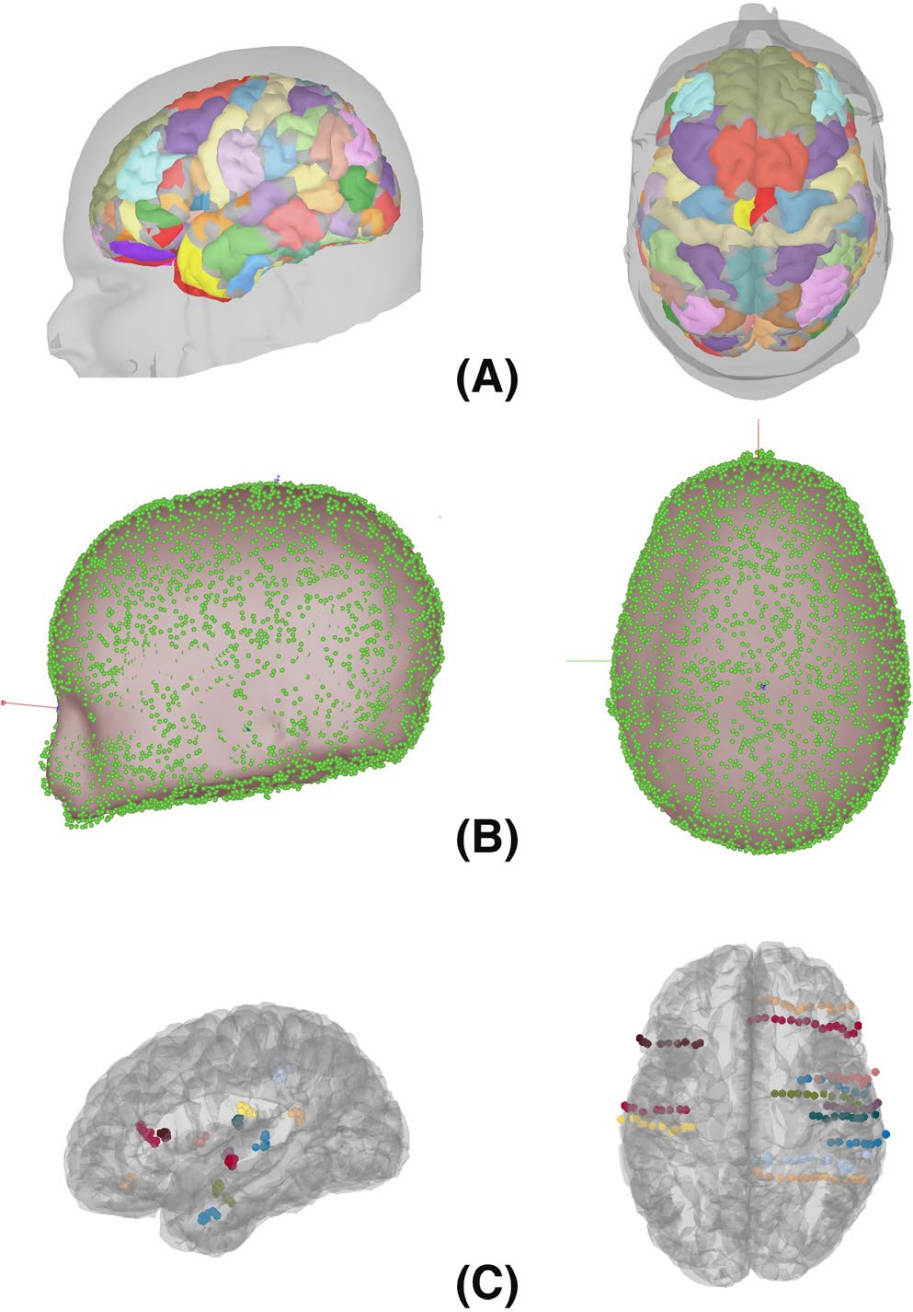
A, Coregistration and mapping of the USCBrain atlas to an individual subject; B, aligning the Curry scalp tessellation (green dots) with the Brainstorm scalp tessellation (gray surface); C, SEEG electrodes for a single subject mapped to the common space atlas brain

An overview of the entire sequence is shown in Figure 1. The sections highlighted as being performed within Brainstorm are fully automated. Step 4 completes within seconds and is a direct replacement for the 3‐ to 4‐hour process of manually labeling the contacts using the coregistered MRI/CT. Illustrations showing atlas‐based segmentation and labeling of the brain, aligning the Curry scalp tessellation with the Brainstorm scalp tessellation, and finally contact locations mapped from subject space into atlas space are shown in Figure 2. Note that the contacts are collinear along the electrode, but after mapping to the common atlas space, the electrodes may appear to bend depending on the degree of nonlinear warping required to match the atlas to the subject and vice versa.

### 2.4 Comparison

The manual clinical labels, even after harmonization, included some that did not always map to a single region in the USCBrain atlas. For example, while a clinical label of “anterior middle frontal gyrus” maps directly to “middle frontal gyrus‐anterior” from the USCBrain atlas, a less specific clinical label, “middle frontal gyrus” could map to either of two subdivisions defined in the USCBrain atlas: “middle frontal gyrus‐anterior,” or “middle frontal gyrus‐posterior.” Either could constitute agreement. We resolved this by using all available labels from the USCBrain atlas, and defining a mapping from the clinical label set to the USCBrain atlas label set as a mixture of one‐to‐one and one‐to‐many. For each SEEG contact, the clinical label is mapped to its equivalent USCBrain atlas label. If there are two possible labels, it is assigned to both. If a match is found with the atlas label(s), this is taken as agreement between the two methods. In the event that the labels do not match, we also check the USCBrain atlas for the labels at the locations of each of the 32 surface points on the sphere representing the contact. If any agree with the clinical again, we take this as a match. The absence of any match is then deemed a disagreement.

### 2.5 Expert review of disagreements

Half of the subjects from each group described below were selected for a manual review of their disagreeing contact labels. The number of contacts reviewed for each subject was weighted according to the overall agreement percentage for that subject, one contact for 90%‐100% agreement, two for 80%‐90% agreement, and three for 70%‐80% agreement, all selected at random. Two experts (JGM and DRN) were presented with images showing the location of each SEEG contact in the brain and were asked to specify its anatomical location. They then determined if their selection agreed with either the clinical or the atlas‐based label, but were blinded as to which was which. The resulting match, or lack of one, was recorded for each contact viewed.

## 3 RESULTS

The 103 subjects had a total of 17 003 contacts that were analyzed. There were 5 subjects in the 90%‐100% agreement range, 60 in the 80%‐90% range, and 38 in the 70%‐80% range. No subject yielded less than 70% agreement, and the overall average agreement was 82%. The complete results are available in Table A1 in the Appendix 1.

In total, the experts reviewed 120 contacts. In 48% of the cases, the experts agreed with the USCBrain label; in 36% of the cases, they agreed with the clinical label; and in 16% of the cases, they disagreed with both labels, deeming a third option to be a better choice. Each of the labels that the experts disagreed with was then categorized as being either in an adjacent region, or alternatively as a gross error. Of the 62 USCBrain labels examined for disagreement, 61 of them were in an adjacent region, with only a single gross error. Of the 77 clinical labels examined, 72 were in an adjacent region, with only 5 gross errors.

A summary of the gross errors is shown in Table 1. While #1 appears to be a true misidentification, #2 and #3 represent such large errors that these appear to be typos when the fellows were recording locations, possibly with “SFG” entered instead of “STG” and “MTG” instead of “MFG,” respectively. Similarly, #4 was labeled by the fellow as “PO,” which was more likely intended to represent “pars opercularis” than “parieto‐occipital fissure,” and #5 labeled as “IF” for “interhemispheric fissure” rather than “inferior frontal.” While these types of errors would likely be recognized and corrected during presentation at a patient manage conference, the procedure used here for comparison only found these errors once analysis was completed. While this could be viewed as a limitation of our analysis, it is also clearly preferable that errors of this type are avoided rather than corrected after the fact. This in turn highlights one of the benefits of our automated labeling method. The single identified “gross error” associated with atlas‐based labeling arose from a determination of the electrode being in the inferior temporal sulcus when the expert review determined the location to be in the superior temporal sulcus. In fact, the electrode was deep in the superior temporal sulcus, relatively close to the depths of the inferior temporal sulcus.

**TABLE 1:** List of anatomical labels classified as gross errors as part of the expert review

#	Group	Incorrect label	Correct label
1	Clinical	Middle frontal gyrus	Precentral gyrus
2	Clinical	Superior frontal gyrus	Superior temporal gyrus
3	Clinical	Middle temporal gyrus	Middle frontal gyrus
4	Clinical	Parieto‐occipital fissure	Superior temporal gyrus
5	Clinical	Inferior frontal gyrus	Precuneus
6	USCBrain	Inferior temporal sulcus	Superior temporal sulcus

Thirteen of the subjects had a prior resection which was visible in the MRI used for coregistration to the atlas and electrode labeling. The average agreement between the clinical and USCBrain labels was also 82% for these subjects, suggesting that prior resection does not negatively impact the accuracy of the labeling process. A two sample t‐test did not indicate a difference in mean accuracy between subjects with and without prior resection (*P* =.852).

**FIGURE 3:**
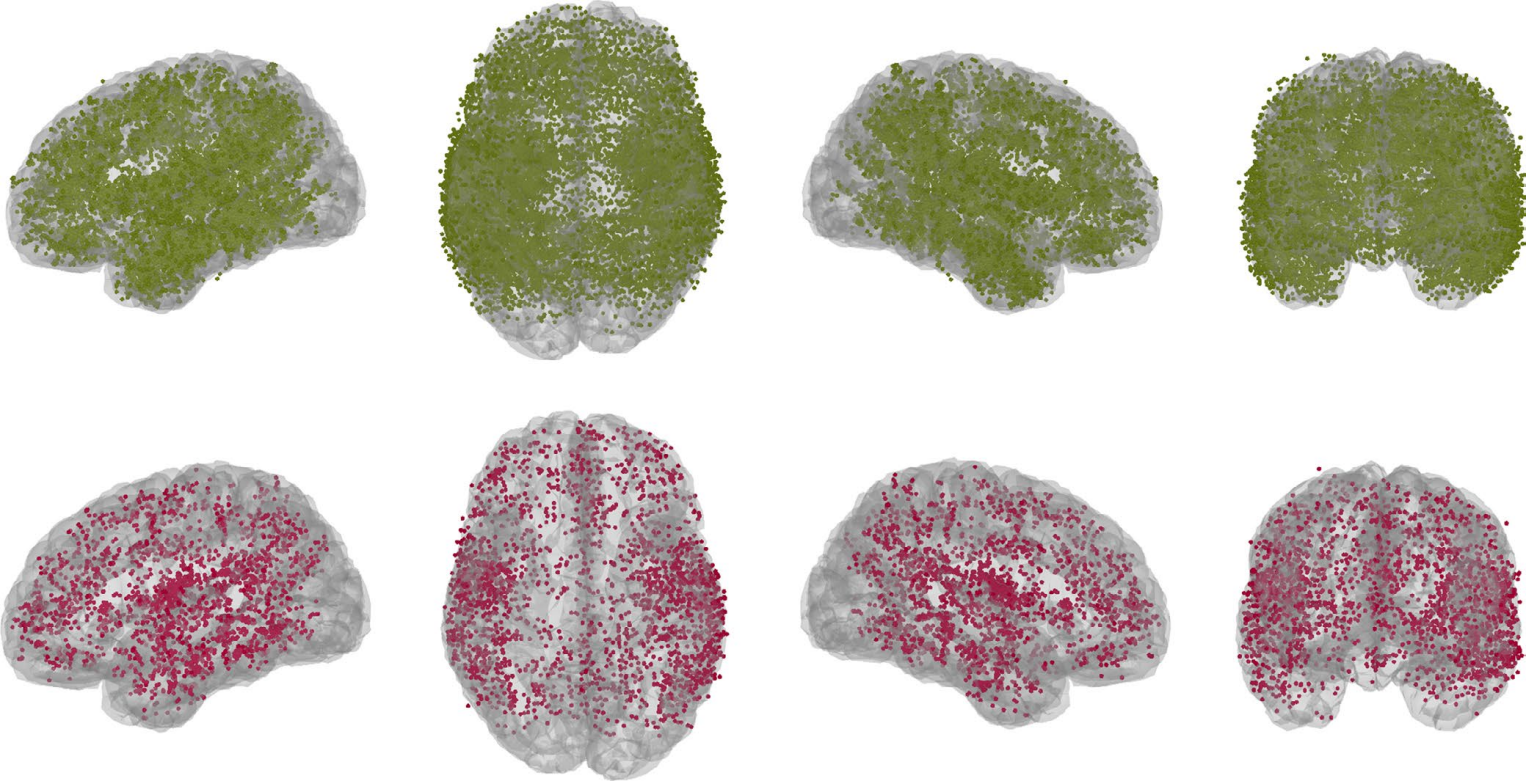
The 17 003 contacts shown as agreeing (green) and disagreeing (red) when mapped to a common space

For the purposes of cross‐subject comparison using the atlas, we used the inverse of the atlas‐to‐subject mapping to map the contact locations for each patient to the common atlas space. This map is automatically computed as part of the BrainSuite registration process. Figure 3 shows the map of which contact labels agreed and disagreed between clinical and atlas‐based labeling. Left and right opercular regions seem to show a particularly large degree of disagreement.

We examined the most commonly applied clinical labels and report their respective agreements and disagreements with the atlas‐based labels in Table 2. The three most commonly seen clinical labels which revealed the lowest agreement with atlas‐based labels in Table 2 were the superior temporal sulcus, frontal operculum, and superior frontal sulcus. Table 3 shows a breakdown of the atlas‐based labels applied to these three regions in the event that a disagreement with the clinical label was identified. One instance of a gross error, marked with a *, is clearly evident in the table. It consisted of 7 contacts spread across 2 electrodes for a single subject, all of which were labeled MTG by BrainSuite but SFS clinically. Inspection of the location of the contacts revealed them to indeed be within the MTG, with the most likely explanation for an error of such magnitude being a typo of SFS instead of STS by the fellow rather than misidentification. The typo aside, the remaining labels demonstrate the “adjacent region” nature of the pair of labels as described in the expert review. As the boundaries of sulcal regions are sometimes difficult to define, such differences are not surprising. For example, if a clinical label is within the superior temporal sulcus, it is likely there will be cases where the atlas‐based label is either the superior temporal gyrus or middle temporal gyrus as these are the border the superior temporal sulcus. Similarly, the rostral extent of the STS is bounded by the temporal pole and more medially it abuts the transverse temporal gyrus and so on. Therefore, these “disagreements” are not gross errors and can be explained by individual variations in anatomy, variability in the fellow expertise, and subjective decisions on the location of gyral boundaries.

**TABLE 2:** The most commonly applied clinical labels and the corresponding agreement with USCBrain Disagreements marked with a * are expanded in Table 3. Also included are the various hippocampus and insula labels which highlight their frequency of usage in freeform labeling

Clinical label	Count	Agree	Disagree	Agreement
White matter	3214	3006	208	0.94
Out (not in brain)	719	699	20	0.97
Superior temporal sulcus	556	420	136*	0.76
Frontal operculum	539	405	134*	0.75
Middle frontal gyrus	519	461	58	0.89
Middle temporal gyrus	481	461	20	0.96
Temporal pole	467	455	12	0.97
Supramarginal gyrus	370	307	63	0.83
Superior temporal gyrus	367	325	42	0.89
Amygdala	359	339	20	0.94
Intraparietal sulcus	344	308	36	0.90
Superior frontal sulcus	336	238	98*	0.71
Parietal operculum	333	313	20	0.94
				
Hippocampus	156	143	13	0.92
Hippocampal head	97	95	2	0.98
Hippocampal tail	73	64	9	0.88
Hippocampal body	61	57	4	0.93
Posterior hippocampus	31	25	6	0.81
Anterior hippocampus	14	14	0	1.00
				
Anterior insula	247	201	46	0.81
Posterior insula	233	204	29	0.88
Insula	27	26	1	0.96

**TABLE 3:** Selected labels from Table 2 with relatively high disagreement, showing the BrainSuite labels applied to the center of the contact in each case

Clinical label	STS	136	Frontal operculum	134	SFS*	98
Breakdown of USCBrain labels	MTG	74	Precentral gyrus	64	SFG	48
	STG	43	Circular sulcus	31	MFG	40
	TTG	6	Postcentral gyrus	26	MTG*	7
	Temporal pole	4	Precentral sulcus	4	Precentral gyrus	2
	Out	3	Central sulcus	2		
	ITG	2	Insula	2	Pars triangularis	1
	ITS	2	Sylvian fissure	2		
	Angular gyrus	1	Out	2		
	White matter	1	White matter	1		

Note

Abbreviations: ITG, inferior temporal gyrus; ITS, inferior temporal sulcus; MFG, middle frontal gyrus; MTG, middle temporal gyrus; MTG, middle temporal gyrus; SFG, superior frontal gyrus; SFS, superior frontal sulcus; STG, superior temporal gyrus; STS, superior temporal sulcus; TTG, transverse temporal gyrus.

* Prior resection.

## 4 DISCUSSION

The ability to precisely determine the anatomical location of SEEG electrode contacts is critical for both medical diagnosis and research. In this paper, we present a semi‐automated labeling methodology that had a high degree of concordance with our current clinical workflow. The majority of patients had a range of 80%‐90% agreement between the atlas‐based label and the clinical label with an average of 82% overall. No subject yielded less than 70% agreement. Validation of our method for anatomically labeling SEEG contacts consisted of a retrospective analysis involving comparison against anatomic labeling assigned during a standard clinical workflow, and subsequent expert review of particular disagreeing cases.

In our study, the error rate did not differ substantially in cases where a prior resection had been performed compared to those with no prior surgery. In those contacts where there was disagreement in labeling, the differences were frequently minor, in the sense that the two labels assigned to a given contact were adjacent to each other. Only a small number of gross errors were found, nearly all in the clinical workflow labeling. Of those, most are possibly due to errors in recording data rather than actual misidentification of anatomical structures. Only one atlas‐based label was initially deemed to contain a gross error by expert review. However, on further review of the anatomic location of the electrode the error could in fact be considered an adjacent error.

The semi‐automated process of electrode labeling described here is likely to aid workflow in the analyses of these complex datasets. Each patient reviewed had an average of 170 contacts. This highlights the tedious nature of clinical anatomic review of each individual contact. Those contacts which are of high importance including those in the epileptogenic zone are more likely to undergo further scrutiny through the course of the clinical workflow. Those electrodes outside of the clinical interest may get less attention which could account for some of the errors in the clinical labels.

Sulcal localization of SEEG contacts is important as these may often be involved in epileptic activity or mark boundaries of surgical resection.^26^ The automated process using BrainSuite allows for distinction of where a contact is within the crown of the cortex, white matter, or a sulcus. Each has significance as it relates to interpreting the iEEG waveforms. Complex dipole manifestations of interictal spikes can better be understood by knowing the anatomic correlation to the electrophysiology recorded at these regions.^27^ The ability to see activity at depths of sulci and deep structures such as the insula and cingulate gyrus is one of the strengths of SEEG. The USCBrain atlas provides 26 sulcus regions per hemisphere including regions such as the superior temporal sulcus, interparietal sulcus, and superior frontal sulcus as well as other important regions known to be important structures in patients with medically intractable focal epilepsy in pathologies such as depth of sulcus dysplasia.^28^

Since some electrodes can sit at a boundary, it is important to know if there is any confidence in the location in one area or multiple. Using the automated BrainSuite atlas labeling method, we were able to discern the agreement between the clinical and automated labels which provides an overall confidence of the contact being solidly in one location or perhaps sitting a border of two regions. Each contact in the automated labeling methodology is represented by 32 points on a small sphere, and by assigning a label to each point on the sphere on a contact, a degree of overall confidence of the anatomic label for each contact can be obtained. The list of labels serves then as a double check of the clinical labeling method, and we expect would offer a tool in training fellows who are learning to assign labels.

The USCBrain atlas uses resting fMRI data of multiple subjects from the HCP database to further segment each hemisphere into 130 subdivisions. Since the extent of abnormalities seen in various SEEG contacts has direct impact to the surgical strategy, even in cases with wide spread pathology such as polymicrogyriay,^29^ subdivision of structures could potentially be used to limit the extent of resection based on anatomo‐electroclinical correlation. As areas within the brain are divided into smaller distinct regions, the importance of localizing SEEG contacts with higher precision becomes even more apparent.

## 5 CONCLUSION

The understanding of the anatomical location of an intracranial array is critically important to both clinical and research‐related investigations of patients undergoing epilepsy surgery using an invasive evaluation with SEEG. The anatomo‐electro‐clinical correlation methodology in particular implies that strict anatomic localization is important to understand the extent of the epileptogenic zone which then guides resection strategies. The automated BrainSuite methodology allows a convenient and quick process to assign SEEG contacts to subgyral and sulcal labels. This automated process was found to have good correlation with the clinical labeling process, a manual process that is time consuming and prone to a greater degree of gross errors. The use of this automated process is likely to serve as a double check for the neurophysiologist reviewing the iEEG findings and may also serve as an educational tool for those trainees who are first starting out in SEEG interpretation.

## CONFLICT OF INTEREST

The authors have no conflicts of interest to disclose. We confirm that we have read the Journal's position on issues involved in ethical publication and affirm that this report is consistent with those guidelines.

## APPENDIX 1

**TABLE A1:** Full results on agreement between clinical and USCBrain labels showing the number of agreeing labels, the total number of implanted contacts, and the agreement percentage

Subject ID	Agree	Total	% Agree	Subject ID	Agree	Total	% Agree
1*	71	91	78	53	117	151	77
2*	73	90	81	54	118	149	79
3	76	95	80	55	94	117	80
4	107	132	81	56	141	168	84
5*	95	111	86	57	120	135	89
6	132	177	75	58	125	167	75
7*	127	158	80	59	165	189	87
8*	103	146	71	60	137	168	82
9	129	151	85	61	107	135	79
10	116	141	82	62	138	162	85
11	99	126	79	63	107	122	88
12	134	156	86	64	98	129	76
13*	80	89	90	65	110	128	86
14	144	173	83	66	132	174	76
15	78	99	79	67*	116	136	85
16	143	178	80	68	127	150	85
17	130	146	89	69	104	137	76
18	143	168	85	70	132	160	83
19	129	152	85	71	93	106	88
20	142	163	87	72	133	140	95
21	121	166	73	73	133	157	85
22	101	117	86	74	111	149	74
23	119	134	89	75	85	111	77
24	112	140	80	76	90	113	80
25	126	162	78	77	102	127	80
26*	89	120	74	78	94	104	90
27	124	146	85	79*	108	134	81
28	160	181	88	80	81	94	86
29	152	171	89	81	117	136	86
30	126	157	80	82	96	112	86
31	101	126	80	83	85	97	88
32*	83	89	93	84	134	170	79
33	139	161	86	85	113	148	76
34	128	162	79	86	129	163	79
35	100	126	79	87	133	155	86
36	44	57	77	88	89	112	79
37	130	166	78	89	73	97	75
38	146	184	79	90	89	118	75
39	107	134	80	91	113	126	90
40	91	115	79	92	118	146	81
41	94	113	83	93	61	78	78
42*	128	171	75	94	99	118	84
43*	102	124	82	95	102	116	88
44	76	88	86	96	133	165	81
45	133	167	80	97	148	175	85
46	74	90	82	98	120	149	81
47	141	156	90	99	148	159	93
48*	75	85	88	100	112	131	85
49	119	165	72	101	122	146	84
50	143	179	80	102	113	135	84
51	132	168	79	103	131	170	77
52	143	173	83				

* Prior resection.

## References

[1] Bancaud J, Bonis A, Talairach J, Tournoux P, Szikla G, Saw V, et al The value of stereotaxic functional exploration in the localization of expansive lesions. Rev Obstet Ginecol Venez. 1961;105:219–220. 13864581

[2] Talairach J, Bancaud J, Szikla G, Bonis A, Geier S, Vedrenne C. New approach to the neurosurgery of epilepsy. Stereotaxic methodology and therapeutic results. 1. Introduction and history. Neurochirurgie. 1974;20(Suppl 1):1–240. 4603712

[3] Bulacio JC, Chauvel P, McGonigal A. Stereoelectroencephalography: interpretation. J Clin Neurophysiol. 2016;33(6):503–10. 27918345 10.1097/WNP.0000000000000305

[4] Isnard J, Taussig D, Bartolomei F, Bourdillon P, Catenoix H, Chassoux F, et al French guidelines on stereoelectroencephalography (SEEG). Clin. Neurophysiol. 2018;48:5–13. 10.1016/j.neucli.2017.11.005 29277357

[5] Alarcon G, Guy CN, Binnie CD, Walker SR, Elwes RD, Polkey CE. Intracerebral propagation of interictal activity in partial epilepsy: implications for source localization. J Neurol Neurosurg Psychiatry. 1994;57(4):435–49. 8163992 10.1136/jnnp.57.4.435 PMC1072872

[6] Cvetkovska E, Martins WA, Gonzalez‐Martinez J, Taylor K, Li J, Grinenko O, et al Heterotopia or overlaying cortex: what about in‐between? Epilepsy Behav Case Rep. 2019;11:4–9. 30456171 10.1016/j.ebcr.2018.09.007 PMC6232626

[7] Bartolomei F, Wendling F, Bellanger JJ, Regis J, Chauvel P. Neural networks involving the medial temporal structures in temporal lobe epilepsy. Clin Neurophysiol. 2001;112(9):1746–60. 11514258 10.1016/s1388-2457(01)00591-0

[8] Pittau F, Vulliemoz S. Functional brain networks in epilepsy. Curr. Opin. Neurol. 2015;28(4):338–43. 26110805 10.1097/WCO.0000000000000221

[9] Nair DR, Mohamed A, Burgess R, Luders H. A critical review of the different conceptual hypotheses framing human focal epilepsy. Epileptic Disord. 2004;6(2):77–83. 15246951

[10] Matsumoto R, Kunieda T, Nair DR. Single pulse electrical stimulation to probe functional and pathological connectivity in epilepsy. Seizure. 2017;44:27–36. 27939100 10.1016/j.seizure.2016.11.003 PMC5291825

[11] Matsumoto R, Nair DR, LaPresto E, Najm I, Bingaman W, Shibasaki H, et al Functional connectivity in the human language system: a cortico‐cortical evoked potential study. Brain. 2004;127(10):2316–30. 15269116 10.1093/brain/awh246

[12] Matsumoto R, Nair DR, LaPresto E, Bingaman W, Shibasaki H, Lüders HO. Functional connectivity in human cortical motor system: a cortico‐cortical evoked potential study. Brain. 2007;130(1):181–107. 17046857 10.1093/brain/awl257

[13] Enatsu R, Piao Z, O’Connor T, Horning K, Mosher J, Burgess R, et al Cortical excitability varies upon ictal onset patterns in neocortical epilepsy: a cortico‐cortical evoked potential study. Clin Neurophysiol. 2012;123(2):252–60. 21802356 10.1016/j.clinph.2011.06.030

[14] Dionisio S, Mayoglou L, Cho SM, Prime D, Flanigan PM, Lega B, et al Connectivity of the human insula: a cortico‐cortical evoked potential (CCEP) study. Cortex. 2019;120:419–42. 31442863 10.1016/j.cortex.2019.05.019 PMC6825888

[15] Qin C, Tan Z, Pan Y, Li Y, Wang L, Ren L, et al Automatic and precise localization and cortical labeling of subdural and depth intracranial electrodes. Front Neuroinform. 2017;11:10. 28261083 10.3389/fninf.2017.00010 PMC5314105

[16] Granados A, Vakharia V, Rodionov R, Schweiger M, Vos SB, O’Keeffe AG, et al Automatic segmentation of stereoelectroencephalography (SEEG) electrodes post‐implantation considering bending. Int J Comput Assist Radiol Surg. 2018;13(6):935–46. 29736800 10.1007/s11548-018-1740-8 PMC5973981

[17] Pieters TA, Conner CR, Tandon N. Recursive grid partitioning on a cortical surface model: an optimized technique for the localization of implanted subdural electrodes. J Neurosurg. 2013;118:1086–97. 23495883 10.3171/2013.2.JNS121450

[18] Princich JP, Wassermann D, Latini F, Oddo S, Blenkmann AO, Seifer G, et al Rapid and efficient localization of depth electrodes and cortical labelling using free and open source medical software in epilepsy surgery candidates. Front Neurosci. 2013;7:260. 24427112 10.3389/fnins.2013.00260 PMC3876273

[19] Gonzalez‐Martinez J, Mullin J, Vadera S, Bulacio J, Hughes G, Jones S, et al Stereotactic placement of depth electrodes in medically intractable epilepsy. J Neurosurg. 2014;120(3):583–790. 24405074 10.3171/2013.11.JNS13635

[20] Shattuck DW, Leahy RM. BrainSuite: an automated cortical surface identification tool. Med. Image Anal. 2002;8(2):129–42. 10.1016/s1361-8415(02)00054-3 12045000

[21] Joshi AA, Choi S, Chong M, Sonkar G, Gonzalez‐Martinez J, Nair D, et al A hybrid high‐resolution anatomical MRI atlas with sub‐parcellation of cortical gyri using resting fMRI. bioRxiv. 2020;09(12):294322. 10.1016/j.jneumeth.2022.109566 PMC9302382 35306036

[22] Joshi AA, Shattuck DW, Thompson PM, Leahy RM. Surface‐constrained volumetric brain registration using harmonic mappings. IEEE Trans Med Imaging. 2007;26(12):1657–69. 18092736 10.1109/tmi.2007.901432 PMC4516139

[23] Tadel F, Baillet S, Mosher JC, Pantazis D, Leahy RM. Brainstorm: a user‐friendly application for MEG/EEG analysis. Comput Intell Neurosci. 2011;2011:1–13. 10.1155/2011/879716 21584256 PMC3090754

[24] Joshi AA, Choi SY, Shattuck DW, Leahy RM. A method for automatic demarcation of sulcal and gyral regions on the cortical surface. OHBM; 2019.

[25] Rousseeuw PJ. Silhouettes: a graphical aid to the interpretation and validation of cluster analysis. J Comput Appl Math. 1987;20:53–65.

[26] Isnard J, Taussig D, Bartolomei F, Bourdillon P, Catenoix H, Chassoux F, et al French guidelines on Stereoelectroencephalography (SEEG). Neurophysiol Clin. 2018;48(1):5–13. 10.1016/j.neucli.2017.11.005 29277357

[27] Merlet I, Gotman J. Reliability of dipole models of epileptic spikes. Clin Neurophysiol. 1999;110(6):1013–28. 10.1016/s1388-2457(98)00062-5 10402088

[28] Harvey AS, Mandelstam SA, Maixner WJ, Leventer RJ, Semmelroch M, MacGregor D, et al The surgically remediable syndrome of epilepsy associated with bottom‐of‐sulcus dysplasia. Neurology. 2015;84(20):2021–8. 25888556 10.1212/WNL.0000000000001591 PMC4442099

[29] Maillard LG, Tassi L, Bartolomei F, Catenoix H, Dubeau F, Szurhaj W, et al Stereoelectroencephalography and surgical outcome in polymicrogyria‐related epilepsy: a Multicentric Study. Ann Neurol. 2017;82(5):781–94. 10.1002/ana.25081 29059488

